# Correction: The impact of the COVID-19 pandemic on maternal and perinatal health: a scoping review

**DOI:** 10.1186/s12978-023-01575-2

**Published:** 2023-03-30

**Authors:** Bethany Kotlar, Emily Michelle Gerson, Sophia Petrillo, Ana Langer, Henning Tiemeier

**Affiliations:** 1 Harvard T.H. Chan School of Public Health, Boston, MA USA; 2 George Washington University, Washington, DC USA; 3 Brown University, Providence, RI USA; 4 Department of Social and Behavioral Science, Harvard T.H. Chan School of Public Health, 677 Huntington Ave, Boston, MA 02115 USA

**Correction: Reproductive Health (2021) 18:10** **https://doi.org/10.1186/s12978-021-01070-6**

After publication of this article [1], the author Emily Gerson asked to change her name from Emily Gerson to Emily Michelle Gerson, because there has been confusion regarding publications from other authors named Emily Gerson.

The original article [1] has been corrected.
